# System training and assessment in simultaneous proportional myoelectric prosthesis control

**DOI:** 10.1186/1743-0003-11-75

**Published:** 2014-04-28

**Authors:** Anders L Fougner, Øyvind Stavdahl, Peter J Kyberd

**Affiliations:** 1 Department of Engineering Cybernetics, Norwegian University of Science and Technology, Trondheim, Norway; 2 Institute of Biomedical Engineering, University of New Brunswick, Fredericton, NB, Canada

**Keywords:** Electromyography, Estimation, Myoelectric control, Proportional control, Prosthesis guided training, Prosthetics, Prosthetic hand

## Abstract

**Background:**

Pattern recognition control of prosthetic hands take inputs from one or more myoelectric sensors and controls one or more degrees of freedom. However, most systems created allow only sequential control of one motion class at a time. Additionally, only recently have researchers demonstrated proportional myoelectric control in such systems, an option that is believed to make fine control easier for the user. Recent developments suggest improved reliability if the user follows a so-called prosthesis guided training (PGT) scheme.

**Methods:**

In this study, a system for simultaneous proportional myoelectric control has been developed for a hand prosthesis with two motor functions (hand open/close, and wrist pro-/supination). The prosthesis has been used with a prosthesis socket equivalent designed for normally-limbed subjects. An extended version of PGT was developed for use with proportional control. The control system’s performance was tested for two subjects in the Clothespin Relocation Task and the Southampton Hand Assessment Procedure (SHAP). Simultaneous proportional control was compared with three other control strategies implemented on the same prosthesis: mutex proportional control (the same system but with simultaneous control disabled), mutex on-off control, and a more traditional, sequential proportional control system with co-contractions for state switching.

**Results:**

The practical tests indicate that the simultaneous proportional control strategy and the two mutex-based pattern recognition strategies performed equally well, and superiorly to the more traditional sequential strategy according to the chosen outcome measures.

**Conclusions:**

This is the first simultaneous proportional myoelectric control system demonstrated on a prosthesis affixed to the forearm of a subject. The study illustrates that PGT is a promising system training method for proportional control. Due to the limited number of subjects in this study, no definite conclusions can be drawn.

## Background

Since the 1950’s, proportional control has been a popular topic in research on powered upper limb prostheses. Through a review of this research [1] it was revealed that methods for system training, both the choice of method and the composition of the training data set, need further research in order to achieve acceptable results with proportional myoelectric control. Proportional control is currently available as an option from all manufacturers of commercial myoelectric prostheses, but not yet for *simultaneous* control of multiple motor functions.

Proportional control allows for small, precise movements as well as rapid, coarse movements. This can be a useful property for a prosthesis system, and it is hypothesized that it will be useful also for multifunction prostheses. It is also hypothesized that proportional control will enhance the user’s control ability significantly because a continuous relationship between muscular contractions and prosthesis response will allow for more rapid and high-fidelity corrections of movements that deviate from the user’s motor intent.

The conventional method for proportional control of multifunction myoelectric prostheses is *sequential control*, with detection of co-contractions of antagonist muscles for switching between functions [1,2].

Some authors have studied the estimation of multiple forces/torques or positions/angles, with the intention of using the estimates as simultaneous proportional control setpoints, but so far these methods have not been implemented in actual multifunction prostheses [1,3-8].

Historically, testing of pattern recognition systems has relied on the publication of percentage scores of success. This is not a sufficient metric for the utility of pattern recognition in real prostheses. More recently, some research groups have begun to use scores for simulated activities [9,10]. However, since the motion of the prosthesis and socket has an adverse effect on the myoelectric signals [11-14], abstract trials are not sufficient for testing the practicality of a pattern recognition scheme. Tests based on activities that represent real use are more useful. Critically, the choice of the appropriate test is important and an initiative by a body of professionals (ULPOM - Upper Limb Prosthetics Outcome Measures group) [15] has used the WHO-ICF model to define the domains of competence for different tests and identified those tests with sufficient psychometric properties to make valid assessments of prosthesis function [16,17].

This paper presents a novel method for simultaneous proportional control of two motor functions. It has been adapted to a commercially available prosthesis hand and wrist rotator. A system training method was developed based on *prosthesis guided training*[18,19], extended to be used for proportional control. Using the WHO-ICF model, assessment methods were chosen to test normally-limbed subjects with practical tasks in the Function and Activity domain. In order to do that, a prosthesis socket for normally-limbed subjects was designed specifically for the chosen system training method.

Systematic testing of four control schemes has been performed. This includes a traditional control method (sequential control, where switching is performed by co-contractions), a modern pattern recognition system with mutex on-off control, and a method for mutex proportional control.

## Methods

### Test subjects

As described in the “Control system assessment in the function and activity domains” section, the data collection for assessment of all four control strategies was a time-consuming process lasting for several weeks per subject. The study was conducted with two normallylimbed subjects, in order to demonstrate the viability of the system before involving prosthesis users.

Both subjects were right-handed males, age 27 and 30 years. Neither of the subjects had any previous experience with using a prosthesis, but both were familiar with electromyography and prosthesis control technology in general and the research project in particular. Informed written consent was obtained from both participants, and the experimental protocol was approved by the Regional Ethical Committee (2012/1754/REK midt).

### Sensors and actuators

Wireless Trigno electrodes (Delsys Inc., Boston, MA, USA) were used for recording of electromyographic (EMG) signals [20]. These are bipolar with an inter-electrode distance of 10 mm.

The prosthesis consisted of a Motion Control Hand with a brushless DC motor option, and a Motion Control Wrist Rotator (Motion Control Inc., Salt Lake City, UT, USA). The prosthesis was covered with a silicone glove.

The control system was implemented on a computer using LabView, Matlab and a National Instruments wireless data aquisition (DAQ) module.

### Socket design

**Figure 1:**
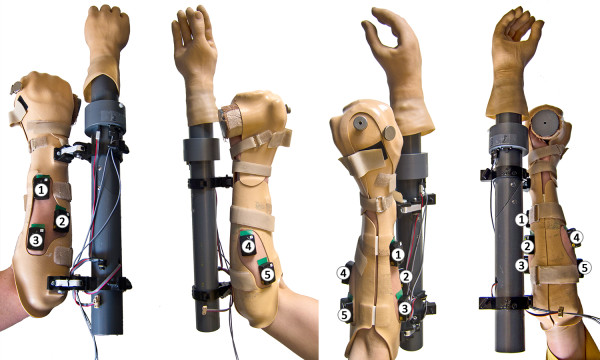
Socket design and electrode placements demonstrated on one of the subjects (lateral, medial, top and bottom view, respectively).

The socket and the electrode placements are shown in Figure 1. The prosthesis socket was designed for normally-limbed subjects, inspired by previous designs by Kyberd [21] and Bouwsema [22], and adjusted to the use of proportional myoelectric control of multiple motor functions. In order to simulate an amputation and achieve approximate isometric contractions, the prosthesis socket was fit around the subject’s arm, wrist and hand while the hand was gripping a plastic cylinder. A strong and stiff socket material (Otto Bock 617H21 Orthocryl Sealing Resin with 617P37 Hardener Powder) was used to lock the subject’s wrist and hand. Two cut-outs were made for electrode sites. The socket was split along ulna and radius and the edges were reinforced with fiberglass. Stainless steel plates were laminated into the socket in suitable positions and used as fixing points for the gripping cylinder and for the prosthesis.

A similar socket design has previously been demonstrated by Simon [23] for use with higher-level prostheses (upper arm or shoulder level). Their design may have enforced near-isometric contractions, although this was not mentioned or highlighted by the authors.

The prosthesis was fit on a hollow plastic cylinder fixed to the lateral side of the socket with hinged pipe supports. The prosthesis was placed approximately 18 centimeters distal to the normal hand in order to be able to pick up small objects from a table, as well as having the prosthesis visible to the subject, since this was found to be important in the practical testing (described in the “Control system assessment in the function and activity domains” section).

### Electrode placement and EMG preprocessing

Five EMG electrodes were used in this study; three on the lateral side and two on the medial side, as shown in Figure 1. The electrodes were placed on: 

1. m. abductor pollicis longus

2. mm. extensor digitorum & extensor digiti minimi

3. mm. extensor carpi radialis longus & brevis

4. mm. flexor carpi radialis & flexor digitorum superficialis

5. m. pronator teres

The locations were initially found by palpation and confirmed by performing contractions while looking at the EMG signals. Electrodes were fixed using a 4-slot double-sided adhesive skin interface from Delsys. For one of the control methods, only a subset of the electrodes were used (see the “Sequential proportional control” section).

EMG signals were sampled at 2 kHz and segmented to 100 ms windows, which by Farrell *et al.* has been reported to be the optimal window length for multifunction prostheses [24]. A set of four EMG features were extracted: Average amplitude value (AAV), zero crossings (ZC), waveform length (WL) – these three are all part of Hudgins’ feature set [25] – and myopulse percentage rate (MYOP) [26,27]. A myopulse output is defined as 1 when the absolute value of the EMG signal exceeds a treshold value (set to 0.009 V for Trigno electrodes with standard settings), and as 0 otherwise. MYOP is the average value of the myopulse output. This feature was found to perform well in pilot studies and was thus included in the feature set. One of the control methods did not use these features (see the “Sequential proportional control” section).

### Intent interpretation and activation profiles

#### Simultaneous proportional control

**Figure 2:**
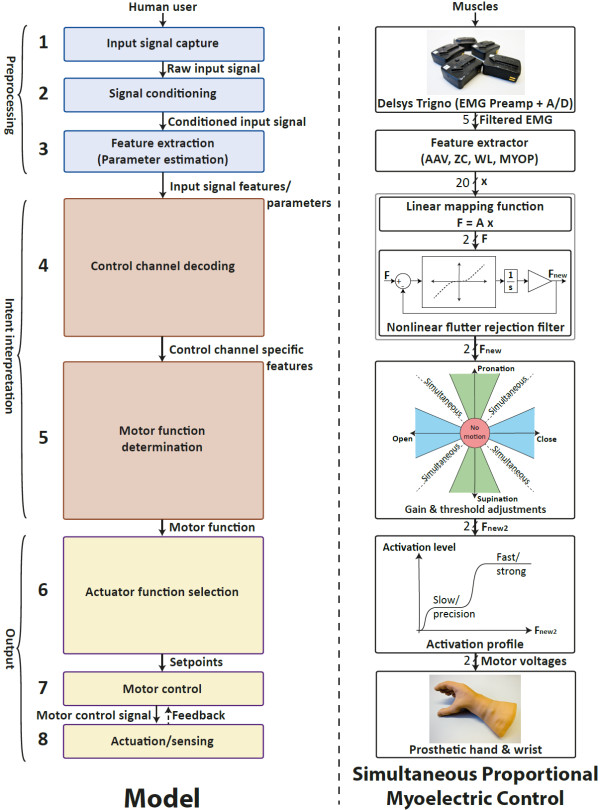
**Control system structure for simultaneous proportional control.*Left:*** Model and taxonomy for the prosthesis control problem [1]. ***Right:*** Control system structure for simultaneous proportional control. The EMG features used are Average amplitude value (AAV), zero crossings (ZC), waveform length (WL) and myopulse (MYOP). In the “gain and threshold adjustments” block, the two axes are spanned by the preliminary activation levels, and the colored sections represent the following: a) Red inner circle: No motion. b) Green sections: Pronation/supination only. c) Blue sections: Open/close only. d) White sections: Pronation/supination and open/close, simultaneously. The “Simultaneous proportional control” section of the paper follows the sequential order of this figure.

Figure 2 shows an overview of the control structure used for simultaneous proportional control. Following the same order: The mapping function is linear and the collection of training data is described in the “Proportional prosthesis guided training” section. The linear mapping is found by minimizing the root-mean-square error of the estimate for the training data set. After mapping, there is one stage of nonlinear filtering (the filter design is indicated in the figure), suppressing fast and small-amplitude variations of input to the prosthesis motors. This smoothens the estimate and thereby reduces wear and tear on the motors. The nonlinearity is defined by *y*=|*x*|tanh(*kx*) and is basically a smooth approximation of a dead-band. Pilot studies showed that this filter works better than an ordinary low-pass filter for flutter suppression, by applying heavy smoothing to low-amplitude signal sections while still being transparent to fast variations of significant amplitude. The rationale for performing the flutter rejection on the channel specific features (F) instead of the more abstract raw EMG or EMG features (x) is that F contains the quantities that determine the activation of the different motor functions. Hence, it reduces the flutter here directly, and it predictably influences the smoothness of the control as observed by the user.

For the next stage, “Gain and threshold adjustments”, the figure shows a domain spanned by the preliminary activation levels. The colored areas of this domain will correspond to the following motor functions: 

● Prosthesis at rest (within the red inner circle).

● Single motor function (green and blue areas; chosen by setting the angles defining their boundaries, hereby called “threshold angles”).

● Simultaneous motor functions with fixed ratio co-activation (white areas).

Threshold angles were individually and manually adjusted at the start of each recording session, to values permitting the user to intentionally and predictably visit all sectors. In the present data they were in the range 18–25 degrees. Gains were adjusted so that the subject is able to reach the maximum motor function activation in all directions by doing maximum voluntary contractions. The adjustment procedure took approximately five minutes. The precise parameter values were not recorded.

Limiting the options to single motor function activation or simultaneous fixed-ratio co-activation makes the prosthesis behave more predictably. This was found to be crucial during initial trials.

The activation profile [1] is generated by using two sigmoid functions on top of each other (as illustrated in Figure 2). This makes it easier to achieve a low speed/low force for precision tasks and a high speed/high force for other tasks. Thus, the system is a hybrid between *multi-level control* and *proportional control*. The activation profile is applied to each of the components of the *F*_*n* *e* *w*2_ signals. Although two distinct activation levels dominate it is still possible to achieve all levels, so it is referred to as *proportional control* as defined by Fougner ([1], see Definition 1 on p. 663, and Fig. two on p. 666). The amount of time spent at each activation level and in each sector of the “gain and threshold adjustments” block was not recorded.

The system training method is explained in the “Proportional prosthesis guided training” section.

#### Mutex proportional control

This system is almost identical to the previous system (Section “Simultaneous proportional control”); the only difference is that simultaneous motions are disabled by setting the “threshold angles” to 45 degrees. This is similar to using an LDA classifier and a speed/force estimator in parallel, as proposed by Hudgins [25].

#### Mutex on-off control

**Figure 3:**
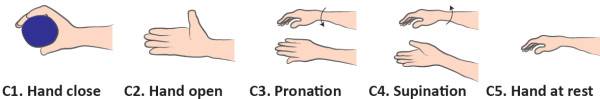
Motion classes used in mutex on-off control.

Five motion classes (C1–C5) were used, as shown in Figure 3. The EMG feature set was classified using linear discriminant analysis (LDA) and the prosthesis output was set to 60% of maximum speed/force for all motions (i.e. C1–C4).

Generally, the training method involved one second of preparation (doing the contraction) and two seconds of sampling (keeping the contraction) for each motion class. During initial trials, PGT was evaluated for mutex on-off control. However, it was unsuccessful because the subjects were supposed to keep the contraction for two seconds, but the prosthesis stopped when reaching the end point after less than 0.5 seconds (already before recording anything). Thus, screen guided training (drawings displayed on the computer screen to guide the subject through a sequence of motion classes) was preferred by everyone testing the system and was used for the LDA classifier in all trials reported in the paper.

**Figure 4:**
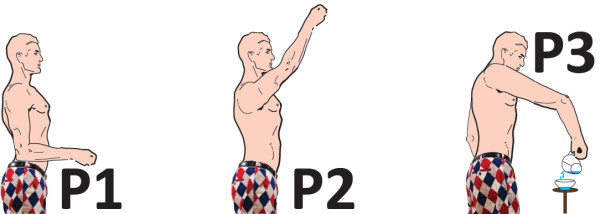
**Limb positions used in system training for simultaneous proportional control, mutex proportional control and mutex on-off control.** Inspired by A. Loomis’ drawings [28].

Each motion class was trained in three limb positions (P1–P3), as shown in Figure 4. Positions P1 and P2 were chosen because it has been shown that it is important to train the control system in a variety of limb positions, especially one with flexed elbow and one with extended elbow [13]. Position P3 was chosen because it appeared during the pilot study that the *water pouring* task of SHAP (see the “Southampton hand assessment procedure (SHAP)” section) was very difficult to perform without training in that limb position.

#### Sequential proportional control

As this was a simulation of conventional control of a prosthetic hand, the two of the Trigno electrodes chosen were; electrodes 2 (finger extensors) and 4 (wrist and finger flexors) shown in Figure 1. As in a conventional system [2], the raw EMG signals were rectified and low-pass filtered, i.e. the EMG features described in the “Electrode placement and EMG preprocessing” section were not used. A differential signal based on the two electrodes was used to control either the hand or the wrist. Co-contractions of antagonistic muscles were detected for switching between the two modes. When the sum of the signals was below some threshold, the prosthesis did not move. As with many commercial systems, the prosthesis defaulted to the ‘hand control’ state at the start of each test.

### Proportional prosthesis guided training

**Figure 5:**
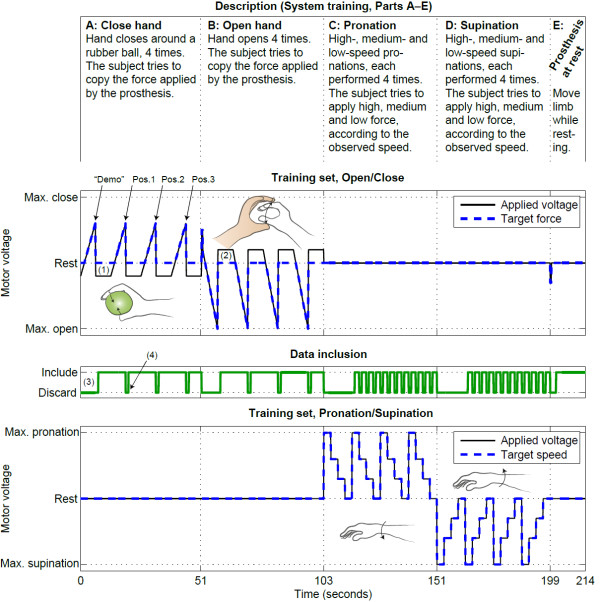
**System training set used for simultaneous proportional control.** The upper plot shows the open/close motor, and the lower plot shows the wrist rotator. Some parts of the training procedure were discarded, as indicated by the boolean variable in the middle plot. The hand (colored) and prosthesis (white) sketches illustrate how each phase of the training was performed. Each motion is repeated four times, as indicated in the figure (“Demo” and Position 1–3; see Figure 3). There are four comments indicated in the figure: (1) “Negative” voltage is needed to open the prosthetic hand between each repetition (closing). (2) “Positive” voltage is needed to close the prosthetic hand between each repetition (opening). (3) First repetition of each activity is a demonstration for the subject and is thus discarded from the recorded data set. (4) After each motor voltage step, one second of the recorded data set is discarded.

The concept of prosthesis guided training has been demonstrated for mutex proportional control by Simon and Lock [18,19]. The procedure was a fixed program (i.e. not influenced by EMG or other user input) demonstrating the intended motions to the subject, and the user was instructed to perform what (s)he perceived as corresponding contractions with muscles in the restricted limb. A similar method for proportional control was developed in the present study. The main difference from previous efforts was that the prosthesis demonstrated continuously varied mechanical properties (e.g. speed or force) instead of a static contraction. Each motor function was trained separately in five parts (A–E), as shown in Figure 5.

For training of hand closing, a rubber ball was placed in the palm of the prosthesis while the hand was closing. The subject observed the compression of the rubber ball and tried to copy the force by using the finger flexors and/or wrist flexors. The motor voltage varied linearly from zero to 60% of maximum force of the prosthesis, i.e. a triangular shape of the motor voltage. In the next phase, hand opening was trained in a similar way. The force was inferred from the opposite hand as it gripped around the prosthesis while it opened.

During initial trials, hand closing was felt by letting the prosthesis grab the subject’s contralateral forearm instead of the rubber ball, thereby offering direct feedback to the subject. However, this could sometimes be painful, and it was found that grabbing a soft rubber ball was more comfortable and practical, especially when training in multiple limb positions. The visual feedback of the ball being squeezed, along with the sound of the prosthesis motors, was found to be sufficient feedback to the subject.

Wrist rotations were trained by observing speed instead of force. In order to make it easier to distinguish the speeds, three distinct values were used; high, medium and low speed. The subjects were instructed to simulate the wrist rotation by only using forearm muscles, i.e. not compensating with the shoulder.

The first four phases of the training were performed four times each. The first contraction of each phase was only for demonstration purposes and was thus not recorded. In the remaining three contractions, the subject was told to keep the arm in the three limb positions (P1–P3) described in Figure 4. It has been demonstrated that this can be useful both for mutex on-off control [13] and for simultaneous proportional control [4]. In the final part, the prosthesis was at rest and the subject was told to let the hand stay relaxed while moving it to the same limb positions.

The total time required for recording the training data set was approximately five minutes, including short breaks between the five parts of the training.

### Control system assessment in the function and activity domains

In order to assess the performance of the control systems, the subjects performed five sessions of test procedures. Within each session, the order of the four control systems was randomized. One session for one control system lasts for 1–2 hours, so the total recording time for each subject was approximately 20–40 hours (during a period of 3–4 weeks).

The following two assessment procedures were used:

#### Clothespin relocation task

The clothespin relocation task originally is a user training method that has more recently been adopted by researchers at the Rehabilitation Institute of Chicago [29-31] for an assessment method. It was chosen in this study because it demonstrates a prosthesis system’s ability to handle a task where at least two motor functions (e.g. hand open/close and wrist pro-/supination) are needed. This test was adopted for the present study. No detailed procedure has yet been published by the team in Chicago, and therefore efforts were made to further standardize the task for future use.

**Figure 6:**
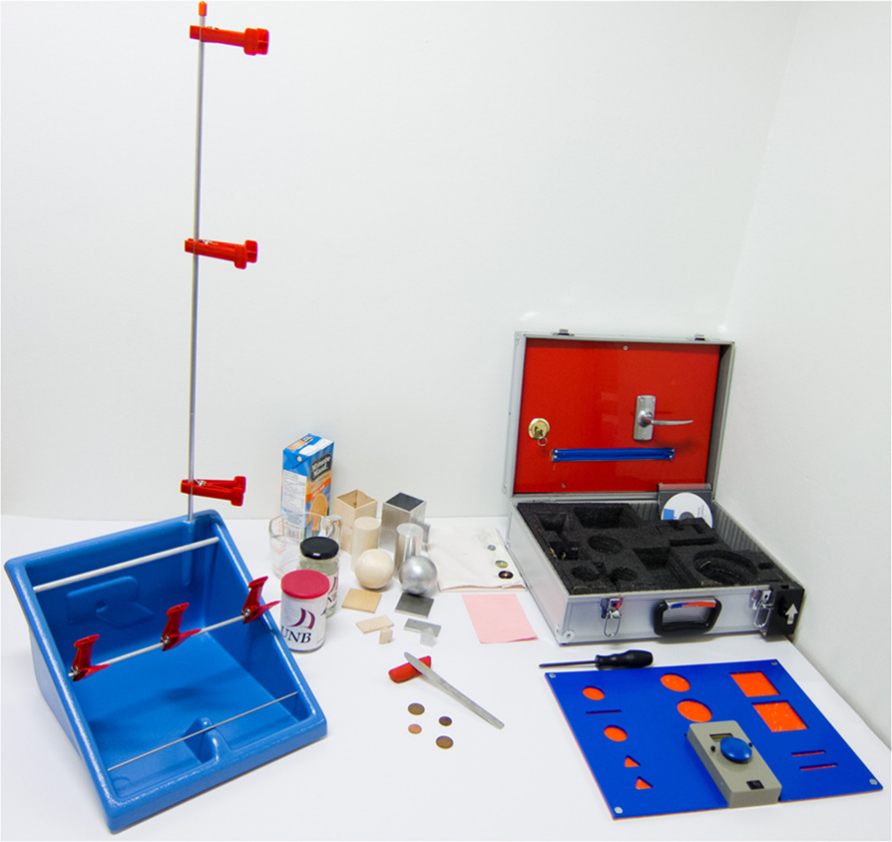
**Equipment used for performance evaluation in the function and activity domains.***Left:* The Original Rolyan Graded Pinch Exerciser with red clothespins. *Right:* The SHAP kit.

Using an *Original Rolyan Graded Pinch Exerciser* with the *red* (2 lbs resistance) clothespins, as shown in Figure 6, and a timer from the SHAP kit, the following tasks are timed: 

● *Up:* Standing in front of the pinch exerciser, with the arm and prosthesis hanging down and the elbow extended, measure the time to move three red clothespins from three positions (left, middle and right) on the middle horizontal bar to anywhere on the vertical bar. The clothespins on the horizontal bar are angled approximately 45 degrees upwards, as shown in Figure 6. The three clothespins are timed individually.

● *Down:* Standing in front of the pinch exerciser, with the arm and prosthesis hanging down and the elbow extended, measure the time to move three red clothespins from three positions (top, middle and bottom) on the vertical bar to anywhere on the middle horizontal bar. The clothespins on the vertical bar are angled approximately 45 degrees towards the hand that is being tested (i.e. the right hand), as shown in Figure 6.

*Timing* is performed by the subject. The subject starts the timer with the unrestricted hand and then starts moving the prosthesis. The subject stops the timer when the clothespin has been released in place. If a clothespin is dropped, restart the timer and the task, but record the failure (unsuccessful attempt). The failed attempts are not taken into account (e.g. as a penalty time), but they are reported along with the Results.

The equipment is placed on a table horizontally aligned with the subject’s hips. The subject is told to keep the feet stationary. Compensatory body movements are permitted, as long as the subject is able to stand without moving the feet.

The trial consists of blocks of moving three clothespins up and down, five times in each session.

#### Southampton hand assessment procedure (SHAP)

SHAP is a clinically validated test of hand function and consists of manipulations on 12 abstract objects (e.g., moving a sphere or a cylinder) and 14 activities of daily living (e.g., using a doorhandle or a zipper, or pouring water). The kit is shown in Figure 6 and is placed on a table horizontally aligned with the subject’s hips. Body movements are not restricted. See [32-34] for a complete description of the procedure.

Each task is self timed and the functional score is based on the task completion time, relative to a normal population. The overall score is out of 100% for the normal population. Scores below 95% imply impairment. The score has been shown to reflect the hand design as well as the control format of the hand. As the subject, the prosthesis and the prosthesis socket remains the same, the score reflects the ease with which the prosthesis is controlled and can thus be used to compare the various control schemes.

SHAP was always performed after the Clothespin Relocation task.

## Results

**Figure 7:**
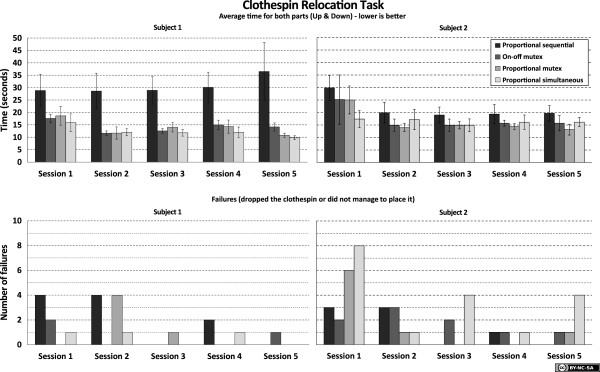
**Results for the Clothespin Relocation task.** Two normally limbed subjects were used. The top charts show results from the Clothespin Relocation Task, where the time represents average time for moving a clothespin up and down (shorter time is better). The error bars show the standard deviation within the session. The bottom charts shows the number of failures/dropped items recorded during each session.

**Figure 8:**
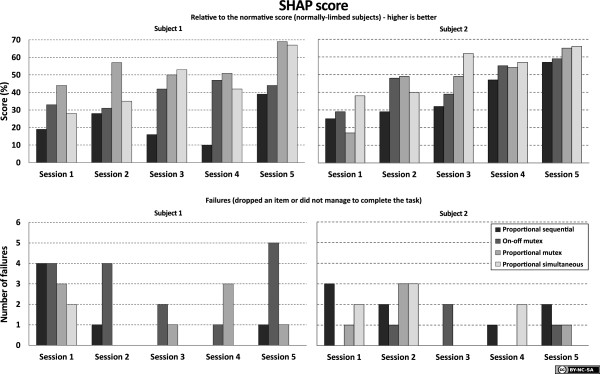
**Results from SHAP.** Two normally limbed subjects were used. The top charts show the SHAP scores, where a higher score is better (100 is the score of the normal population). The bottom charts show the number of failures/dropped items recorded during each session.

The results from the *Clothespin Relocation task* are presented in Figure 7 for both subjects, and results from *SHAP* are presented in Figure 8. It can be observed that the conventional, sequential control method is inferior to the three other methods for these subjects. No significant differences can be found among the other methods, and no significant differences are found in the number of failed attempts.

For the *Clothespin Relocation task*, the results were stable after two to three sessions. This indicates that the subjects had learned both the prosthesis behavior in conjunction with this task, and the task itself, for all four control systems. Subject 1 stabilized at an average completion time of 30–35 seconds for sequential proportional control and 10–15 seconds for the other systems. Subject 2 stabilized on an average completion time of approximately 20 seconds for sequential proportional control and 13–17 seconds for the other systems.

The standard deviation was significantly higher for sequential proportional control than for the other systems. Subject 1 had an increased completion time for sequential proportional control in the last session (from 30 to 36 seconds), but this increase was smaller than the standard deviation (12 seconds) and can thus be ignored.

It was observed that when using sequential proportional control, subjects frequently used *compensatory movements* (such as moving the upper body and using the shoulder joint) instead of wrist rotation during the Clothespin Relocation task.

Regarding the *SHAP* scores, they are not completely stabilized even after the five sessions recorded in this study; so we cannot determine if the subjects have yet completely learned to handle the prosthesis, or the test procedure itself. Nevertheless, the results are consistent in the sense that both subjects initially perform at approximately 20–40% and reach a level of up to 60–70% in the last session. Overall the scores are lower for sequential proportional control than for the other three systems.

## Discussion

The *prosthesis socket developed for normally-limbed subject* (Section “Socket design”) in this study cannot replace the need for testing on prosthesis users, but it is a useful tool for practical tests of prosthesis control systems. Since the socket locks all the joints of the subject’s forearm, hand and fingers, the muscle contractions are approximately isometric. Practical tests, using this socket with a prosthesis, are likely to be more relevant than reports of offline classification (or estimation) error rates on pre-recorded signals from the laboratory, as demonstrated by Hargrove [10] and Fougner [13]. The 18-centimeter extension distally past the hand is a large extension and would have been problematic if SHAP contained tasks such as eating or drinking. However, the added length is not believed to be crucial during SHAP and the Clothespin Relocation task. Similar extensions have been used in previous studies [21,22].

The use of pattern recognition relies on the computer system learning the patterns of activation of the muscles to control the hand. These patterns may not be stable in the short or long term, and this can be the reason for several unsuccessful attempts to create a practical pattern recognition system. The introduction of prosthesis guided training (PGT) [18] is the single largest contribution to the development of a practical control system based on pattern recognition, since it may allow the prosthesis user to re-train a system whenever it does not work satisfactorily. Regarding the stability of the patterns used in this study, it was not measured quantitatively, but no descrease in performance was apparent during each 1-2 hour recording session. PGT was further developed in the present study for use with simultaneous proportional control. The use of a rubber ball (or other tools) enables the prosthesis user to observe the force applied by the prosthesis when closing or opening, rather than just observing the speed. This may be important for proportional control.

For practical reasons, only speed was observed while training wrist rotation. It was found impossible to know whether the motor was told to apply a large or medium force, since the motor stops whenever it meets resistance in order to save battery power. To observe and recognize rotational speed was also difficult, so it was chosen to use three distinct values. For these reasons, and because the reported method was quite time-consuming (approximately five minutes), further development is advised.

This study has demonstrated a proportional version of PGT, using continuously varied contractions for training of proportional control. Although the linear mapping function does not require training at all contraction levels, we believe that a graded contraction may be more robust than fixed-ration contractions since it contains a larger variation in user effort. This is similar to adding more limb positions, dynamic movements or electrode shifts to a training set. Future studies should compare the use of fixed-ratio and graded contractions in PGT.

For practical reasons, screen-guided training (SGT) was used for mutex on-off control in the present study. The essential difference between this method and PGT in the case of mutex on-off control is that PGT allows re-traing of the prosthesis in the field. We therefore believe that there would be no significant difference between the results produced by the two methods in the context of this study.

A *simultaneous proportional myoelectric control system* was developed for multifunction prostheses (Section “Simultaneous proportional control”). Due to the low number of subjects involved in the study, conclusions cannot be drawn about the overall performance of this system. Even so, the results indicate that the three modern systems (simultaneous proportional control, mutex proportional control and mutex on-off control) may all be superior to the conventional, sequential proportional control system in practical use. This can reflect differences in the *Preprocessing layer* (e.g. the extracted feature set and the number of electrodes) or the *Intent interpretation layer* (the sequential control itself) of the control system ([1], see Fig. one on p. 667) .

Subject 1 had a much larger difference between proportional sequential control and the other control strategies than did Subject 2 in both the Clothespin Relocation Task and the SHAP. Their comments have been recorded, and while Subject 1 commented that he used function switching actively, rather than using compensatory movements, Subject 2 commented that he disliked switching so much that he tried to use only one prosthesis function for each task (thereby promoting the use of compensatory movements) rather than switching. These comments are subjective comments but may explain the differences on these two subjects.

Future comparison studies with more subjects or prosthesis users are strongly indicated. Such a study would benefit from using PGT in mutex on-off control, so that the training method is more consistent across the compared methods. For simultaneous proportional control, the amount of time spent at each activation level and in each state (each sector of the “gain and threshold adjustments” block) should be recorded, in order to address whether or not the simultaneous and proportional nature of the controller is being utilized.

Each motor function was trained separately. Simultaneous motions in the training set were tested in initial trials, but it was found difficult to observe speed or force on two simultaneous motions. That part of the training protocol was omitted in order to simplify and speed up the training time.

It has not been documented whether the subjects of this study would prefer fully independent control of two motor functions, but it has previously been documented that prosthesis users have that preference [35]. During initial trials, fully independent control of both motor functions was permitted, but it was then chosen to limit the system to a fixed-ratio co-activation in order to make the prosthesis behave more predictably. The rest of the system was identical in the two cases.

All four control systems were trained in three limb positions selected specifically for the tasks involved. During the initial tests this was found to be crucial, especially for moving down clothespins (in the Clothespin Relocation task) and for pouring water (in SHAP). As previously demonstrated, the control systems may also benefit from additional input from inertial sensors (accelerometers) [13] or other sensor modalities.

Subject 2 recorded more failures when using simultaneous proportional control than the other control strategies in the Clothespin Relocation task. The subject’s response was that he may have intentionally have dropped the clothespin instead of completing the task. This allowed the test to be restarted and so he could achieve a shorter recorded time, despite the fact that he was instructed to prioritize task completion. It is important to stress to the subject the priority of completing the task without failures, rather than completing the task as fast as possible. This highlights the underlying problem with timed tasks, which achieve an objective measure more readily. However, since a prosthesis that is slow would be regarded as a poor solution, speed of execution remains a good measure for the performance of a prosthesis.

During the Clothespin Relocation task the subjects were instructed not to move their feet. The frequent use of *compensatory movements* observed while using sequential proportional control indicates that compensatory movements may still be the fastest way to complete the Clothespin Relocation task for this control system – even though the test is designed to encourage the use of two motor functions. This might indicate a need for other test activities with a stronger dependence on using multiple motor functions, or ones with an explicit restriction of compensatory movements. On the other hand, we cannot deduce from our results that all kinds of training effects had died out by the completion of the fifth session. In particular this goes for subjective properties like perceived functional performance, which, given more user training, might increase the to the point where the subject would instinctively prefer to utilize another prosthesis motor function rather than compensating with other body movements. Assessment of such long-term training effects are outside the scope of the present paper.

We believe that during these trials, more compensatory movements were performed during sequential control than during the other control methods. Future studies should thus contain quantitative measures of these movements, which is a relevant but challenging task and demands special instrumentation. In addition, the test method must be altered so that it measures the performance in the needed way: In some cases it may be important to be able to perform the task without the need for compensation – while in other cases, the speed is more important. Compensations are the result of more limited movement (range or degrees of freedom). While the prosthesis might provide some of the missing motions, it is a trade off between speed and convenience when multiple degrees of freedom are provided. A crucial aspect of the desire to provide multiple simultaneous motor functions for users is to create the ability to be faster and more convenient without using potentially harmful compensation strategies.

Learning to use a prosthesis is a complex process and measuring it requires a range of different tools [36]. Using the WHO-ICF system, the tools chosen tested the Function (Clothespin) and Activity (SHAP) domains. A new subject must become familiar with the means of control, the prosthesis dynamics and the best way to perform the task. All of these are part of the learning and improving of the subjects as they perform the tests. It has been demonstrated by Bongers, Bouwsema *et al.* that gross motor control, such as positioning the arm and prosthesis in space, can be learned quickly, whereas learning to control the pinching force requires more time [37,38].

As the Clothespin Relocation task contains relatively few motion patterns and only one type of objects to grasp, its scores stabilised quickly. SHAP, on the other hand, is designed to measure the functional abilty of the hand and so contains a wider set of motion patterns and objects to manipulate. SHAP was thus measuring the subject’s ability to learn how to use the prosthesis and the control formats and would need a longer time (more than five sessions) to achieve good control and consistent scores in a future comparison study.

## Conclusion

A prosthesis socket equivalent was developed in order to allow normally-limbed subjects to perform practical tests of control systems for upper limb prostheses. The main difference from previous efforts is that it gives near-isometric muscle contractions by locking joints of the subject’s forearm, hand and fingers.

The performance of four different control systems were compared. The main finding was that the three modern systems all performed superiorly to the conventional, sequential proportional control system. However, due to the limited number of subjects in this study, no definite conclusions can be drawn. Furthermore, the results indicated the need for test activities with a stronger dependence on using multiple motor functions rather than compensatory movements.

The study illustrates that prosthesis guided training is a promising system training method for proportional control. It also contains the first simultaneous proportional myoelectric control system demonstrated on a prosthesis affixed to the forearm of a subject, which complements the current research focus on simultaneous control.

## Competing interests

The authors declare that they have no competing interests.

## Authors’ contributions

ALF, ØS and PJK contributed to the conception of the study and study design. ALF collected the data and drafted the manuscript. All authors read and approved the final manuscript.
